# Mixed versus Focused Resistance Training during an Australian Football Pre-Season

**DOI:** 10.3390/jfmk5040099

**Published:** 2020-12-18

**Authors:** Lachlan P. James, Jade Haycraft, Anthony Pierobon, Timothy J. Suchomel, Mark Connick

**Affiliations:** 1 Sport and Exercise Science, School of Allied Health, Human Services and Sport, La Trobe University, Bundoora, VIC 3086, Australia; 2 Institute for Health and Sport (IHES), Victoria University, Melbourne, VIC 3011, Australia; Jade.Haycraft@vu.edu.au; 3 Northern Knights Football Club, Melbourne, VIC 3072, Australia; A.Pierobon@latrobe.edu.au; 4 Department of Human Movement Sciences, Carroll University, Waukesha, WI 53186, USA; tsuchome@carrollu.edu; 5 School of Human Movement and Nutrition Sciences, University of Queensland, Brisbane, QLD 4072, Australia; m.connick@uq.edu.au

**Keywords:** strength, power, countermovement jump, periodisation

## Abstract

The purpose of this investigation was to determine the effect of a focused versus mixed-methods strength-power training plan on athletes undertaking high volumes of concurrent training. Fourteen junior elite male Australian football players were randomly assigned into either the focused or mixed group. Both training groups undertook a sequenced training intervention consisting of a four-week mesocycle emphasising heavy strength followed by a four-week mesocycle of high velocity emphasis. Training differed between groups by way of the degree of emphasis placed on the targeted attribute in each cycle and occurred during the preseason. Testing occurred pre- and post-training and consisted of the unloaded and loaded (+20 kg) countermovement jump (CMJ). Focused training elicited practical (non-trivial) improvements in flight time to contraction ratio (FT:CT) (g = 0.45, ±90% confidence interval 0.49) underpinned by a small reduction in contraction time (g = −0.46, ±0.45) and a small increase in braking (g = 0.36, ±0.42) and concentric phase mean force (g = 0.22, ±0.39). Conversely, the mixed group demonstrated an unchanged FT:CT (g = −0.13, ±0.56). Similar respective changes occurred in the loaded condition. Preferential improvements in FT:CT occur when a greater focus is placed on a targeted physical quality in a sequenced training plan of junior elite Australian football players during preseason training.

## 1. Introduction

Improving measures of athletic performance requires the strategic manipulation of training variables to meet predetermined goals. Of particular relevance to practitioners is the development of strength (i.e., high force) qualities, which are widely acknowledged to underpin success across a range of field invasion [1,2,3] and combat sports [4,5], all with concomitant endurance demands. For the development of rapid force production through to take-off or release (generically referred to as maximal power, impulsive efforts or speed-strength) resistance training must be structured such that doses of heavy load and high velocity stimuli are integrated throughout a training plan to maximize adaptations [6]. It is generally accepted that to optimize the development of high velocity force production a sequential approach to progressing training is preferred [6,7]. In this method, the benefits of previously developed strength qualities potentiate the development of the next attribute. For example, a strength-endurance/muscular hypertrophy mesocycle will provide the necessary cross-sectional area and force production capacity to maximise strength gains [8], while increased strength provides the neuromuscular foundation to better respond to power training [9,10]. In support of this notion, James et al. [9] observed that that stronger participants (one-repetition maximum [1-RM] back squat = 2.0 × body mass [BM]) displayed significantly greater improvements in countermovement jump (CMJ) peak velocity (d = 0.99 vs. d = 0.35) than those of a much lower baseline strength level (1-RM back squat = 1.2 × BM) when exposed to the same heavy and light load ballistic training intervention. These findings establish the importance of a sequenced approach where training is focused in a primary strength quality during organised phases.

Developing expressions of force at high velocities also requires a mixed method approach whereby a degree of variation in the force–velocity characteristics is needed for the greatest improvements to be realized [11,12]. Because the enhancement of rapid force production is multifactorial, maintenance doses of a non-emphasised attribute (i.e., high velocity lifts in a maximal strength mesocycle) are required to minimize the decay of non-targeted components. The absence of adequate variation may therefore attenuate adaptations as the training plan progresses [9,10]. As such, strength and conditioning programs in team invasion sports generally include a range of modalities and loading conditions [13,14]. However, too much variation in the nature of the training stress limits the opportunity to properly develop the targeted quality ultimately impacting long-term adaptations [15]. This creates a conundrum for the practitioner who must decide whether training is highly focused on a single force-velocity attribute, or, alternatively, if a mixed force-velocity stimulus is superior.

In field invasion sports, rapid force production capabilities must often be developed in the presence of other training tasks. For example, training and match-play in Australian football contains a major endurance demand but also requires fast, maximal expressions of force [16,17,18]. Players in the Australian Football League (AFL) accumulate up to 22 km per week during preseason [19] and are exposed to explosive actions such as sprinting, jumping, and high speed collisions [17,18]. Accordingly, measures derived from the countermovement jump (CMJ) have been reported in several studies at both the elite and junior development levels of the AFL pathway, with increasing values occurring as competition standard increases [16,17]. Because of the interference that can occur to rapid force production when extensive workloads are undertaken [20], the ideal distribution (i.e., more focused versus more mixed) of heavy strength and high velocity training is particularly important to ensure positive adaptations occur despite high volumes of concurrent training. 

Therefore, although the optimal development of rapid force production capabilities appears to require a prior enhancement of strength, there must also be a degree of variation in the force-velocity characteristics of the training stimulus. This is further complicated when high volumes of concurrent endurance training are also being experienced by the athlete. Little research exists exploring the manipulation of the force versus velocity dose across a sequenced training plan for the development of maximal power in concurrently training athletes. This absence of information limits the ability for practitioners to design informed training plans for athletes that require enhanced power attributes. It is therefore the purpose of this investigation to compare training with a high focus on strength then velocity actions to training with a greater mix of these tasks, on unloaded and loaded CMJ performance. 

## 2. Materials and Methods 

### 2.1. Subjects

Fourteen junior elite male Australian football players (mean ± SD; age: 17.3 ±0.7 years; height: 185.0 ± 7.07 cm; mass 77.0 ± 6.7 kg) from the same club were included in this investigation. These athletes competed in the State U18 league. Participants provided written informed consent and the study was approved by La Trobe University Science, Health & Engineering College Human Ethics Sub-Committee (Ethics Approval Number: HEC18457, 18 December 2018).

### 2.2. Design

A repeated measures within-subject design was used to investigate the effect of two training interventions on CMJ measures. Participants were tested pre and post 8 weeks of training. Training was divided into two, four-week mesocycles to enable a sequenced progression through training for both groups. Training interventions differed by way of the degree of emphasis placed on heavy load and high velocity lifts in mesocycle 1 and 2, respectively.

### 2.3. Training Intervention

The study was undertaken during an 8-week period in the preseason of the State U18 Australian football competition. Participants were randomly assigned into either the focused (*n* = 7) or mixed (*n* = 7) group. Both training groups undertook a sequenced periodized training intervention consisting of a mesocycle emphasising heavy strength followed by a mesocycle of high velocity emphasis. The training program differed between groups by way of the degree of emphasis placed on the targeted attribute in each cycle. For example, the focused group undertook a greater volume of heavy strength in the first mesocycle, whereas the mixed group undertook a greater volume of heavy strength in the second mesocycle when compared to the focused group. However, the primary training stimulus for each mesocycle was the same between groups (i.e., heavy strength then high velocity). Table 1 and Table 2 presents the training plan for both groups. These training sessions occurred prior to field based tasks, and all sessions were monitored by the club’s strength and conditioning coach alongside support staff. During this period, players undertook their usual preseason training activities with the club accumulating the following mean (±SD) distances per week: mesocycle 1 = 22.3 ± 3.8 km/week (6.2 ± 1.5 km/week above 4 m/s); mesocycle 2: 21.4 ± 7.6 km/week (5.2 ± 2.1 km/week above 4 m/s).

### 2.4. Testing

Testing occurred pre- and post-training and consisted of the CMJ unloaded and the CMJ with an absolute load of 20 kg on a force platform (ForceDecks Model FD4000; NMP Technologies Ltd., Vald Performance, Newstead, Queensland, Australia). Prior to testing, the athletes performed a dynamic warmup consisting of squatting and lunging patterns followed by several submaximal jumps at progressively increasing intensities. For the test, participants performed three non-continuous CMJs for maximal height using a self-selected countermovement depth. Each jump was separated by approximately 5 s or until a stable weighting period was established. Hands were positioned on the hips during the unloaded condition while participants grasped a standard Olympic barbell positioned in a high bar position in the alternate condition. Testing was undertaken at the beginning of training prior to commencement of primary activities. 

Jumps were recorded at 1000 Hz via native software (NMP ForceDecks v1.2.6780, Vald Performance, Newstead, Queensland, Australia). This propriety software determined jump initiation as a 20 N change in BW and commenced integration from this point to establish a velocity-time curve. Contraction time was the duration between jump initiation and take-off. Concentric phase duration was calculated between zero velocity and take-off while the eccentric duration was the difference between contraction time and concentric duration. Furthermore, between minimum force and the end of the eccentric phase is considered the eccentric braking phase. In addition to phase durations, specific variables were then extracted from the CMJ with a view to explain changes in jump performance. Flight time to contraction time ratio (FT:CT) was considered the primary outcome variable in each condition as it is a preferred measure of neuromuscular function in these athletes [21,22] and scales the movement outcome to the movement time (flight time divided by contraction time) therefore describing rapid force production capabilities. Peak velocity was included as it represents the output of the jump and is less susceptible to error than jump height derived from force-time data. The relative mean force (N/kg) over the eccentric braking phase, and over the concentric phase were also extracted as they describe the input by the athlete into the action over both phases. The mean of each variable across the three jumps in each condition was brought forward for statistical analysis. 

### 2.5. Statistical Analysis

Hedge’s g effect size [23] with 90% confidence intervals was used to quantify the magnitude of change following training in both groups. The following descriptors were used to interpret these effect size: <0.20 = trivial, 0.21–0.60 = moderate, 0.61–1.20 = large, 1.21–2.0 = very large [24]. Therefore, a non-trivial difference was considered a practical change. All statistical analyses were performed in MATLAB (R2019b, The Mathworks, Inc., Natick, MA, USA). Effect size (g) data are presented ± 90% confidence interval. Descriptive data are presented as mean ± SD.

## 3. Results

### 3.1. Unloaded Countermovement Jump

**Figure 1:**
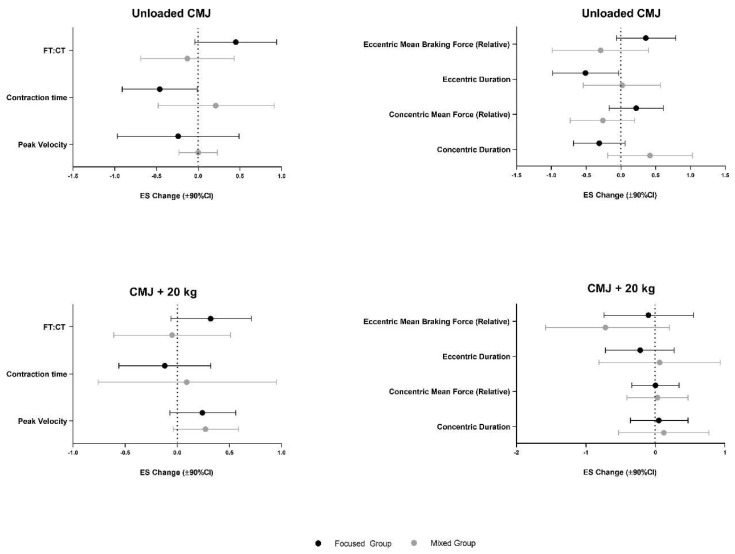
Training induced changes in both the Focused (black) and Mixed (grey) groups across several unloaded and loaded (+20 kg) countermovement jump (CMJ) variables. The magnitude of change is quantified via Hedge’s g effect size with 90% confidence intervals.

The coefficient of variation for unloaded CMJ metrics ranged from 2.5–7.8%. The focused group displayed non-trivial practical changes in all unloaded metrics (Table 3). This included an increase in FT:CT (g = 0.45 ± 0.49) and mean force in both the eccentric (g = 0.36, ± 0.42) and concentric (g = 0.22 ± 0.39) phases alongside decreases in phase durations and peak velocity (Figure 1). In contrast, the mixed group did not improve FT:CT (g = −0.13, ± 0.56), practically increased concentric phase duration (g = 0.42, ± 0.61) and displayed a small practical reduction in mean force over both phases (Figure 1).

### 3.2. Countermovement Jump +20 kg

Measures derived from this condition achieved a coefficient of variation of between 3.2% and 9.7%. Improvements in FT:CT were demonstrated by the focused group (g = 0.32, ± 0.39) accompanied by small improvements in peak velocity (g = 0.24, ± 0.32) and a faster eccentric duration (g = −0.22, ± 0.49) (Figure 1). The mixed group maintained force production and duration throughout the concentric phase but had a notable decrease in eccentric mean braking force (g = −0.72, ±0.89) and unchanged FT:CT (Figure 1). Table 4 presents loaded CMJ variables before and after training across both groups.

## 4. Discussion

The results of this study demonstrate that preferential improvements in rapid force production (represented by the metric FT:CT) occur when a greater degree of emphasis is placed on a targeted physical quality in a sequenced training plan of junior elite Australian football players. The focused group displayed practically meaningful improvements across several CMJ variables in both unloaded and loaded conditions, however, very few positive changes were revealed in the mixed group. These findings indicate the potential advantages of more focused resistance training interventions while highlighting the complexities of developing fast force capabilities in the presence of high volumes of conditioning and sports specific training during the preseason in Australian football. 

The focused group demonstrated practical decreases in unloaded CMJ phase durations alongside increased mean force in both phases. This resulted in the improved FT:CT, albeit with a reduced peak velocity due to the faster movement time limiting the opportunity to accrue impulse during the action. Conversely, the mixed group increased the time spent in the concentric phase a demonstrated reductions in mean force across both subphases, ultimately resulting in an unchanged FT:CT. Similar findings were noted in the loaded condition with FT:CT improving in the focused athletes while it remained unchanged in mixed group. Although no notable changes were observed in the timing or magnitude of force application when examined within phases, collectively the focused group attained an increased peak velocity which underpinned the improved FT:CT result, rather than a faster movement time. The notably reduced braking force displayed by the mixed group in the +20 kg condition was not enough to prevent an increase in peak velocity; however, ultimately this did not translate into an improved FT:CT. Because FT:CT scales movement outcome to movement time, these findings indicate that the focused approach was particularly impactful on the development of rapid force production. Such findings are similar to reports of stronger individuals displaying primary improvements in the timing of force production after exposure to ballistic power training [9,25], and indicates that greater strength might have been developed in the first mesocycle. The ability to produce force rapidly is a key mechanical characteristic in Australian football as it drives acceleration, change of direction, and sprinting to allow the evasion of opposition players or gaining distance when attacking the football [17]. Furthermore, explosive force may be more important for key position players such as rucks, full forwards, and full backs, as any such advantages possessed by these players may determine whether they gain possession of the ball over their opponents [26]. 

These findings provide support for block periodization where a larger emphasis on the development of one quality potentiates the development of the next [8]. Although the mixed group was also sequenced to enable phase potentiation, the greater amount of heavy strength training in the first mesocycle (or ‘block’) likely led to a greater improvement in maximal strength. This would have then enabled superior adaptations to the higher velocity training experienced in the second block [9]. Although the benefits of a combined force-velocity stimulus are well documented, practitioners must find a balance between this variation and an adequate focus that enables an attribute (e.g., maximal strength) to be well developed. The degree to which this balance must occur will be primarily influenced by the existing physiological characteristics of the athlete. Those who are stronger, with a greater resistance training experience will display a more specific adaptation to a given stimulus [9,10]. Therefore, greater variation is needed to target the distinct strength qualities (i.e., maximal strength, RFD under heavy and light loads, etc.) required of performance. In contrast, developmental athletes or weaker individuals, like those in this present investigation, will benefit from a more focused approach. 

The high volumes of concurrent training experienced by the players (22–23 km/week, 5–6 km/week above 4 m/s) is characteristic of an Australian football preseason and certainly interfered with improvements in the CMJ [19,27]. Absence of improvements to CMJ performance metrics during preseason training has been reported previously in elite Australian football [18] and elite rugby union [28], while these measures often peak several weeks into the competitive season [18]. It is notable that maximal strength will increase over the preseason [28] and likely reflects the training objective of the period and the reduced sensitivity to fatigue when compared to high velocity qualities. These findings highlight the challenges of preparing rapid force production for a long competitive season during periods of high aerobic and collision demands. 

FT:CT is considered a desirable metric for the assessment of neuromuscular fatigue in Australian football players [21]. It may be such that the focused group experienced less fatigue following the second mesocycle (due to more high velocity actions and less heavy strength lifts compared to the mixed group) and therefore were better able to express their adaptations. In contrast, the mixed group may experience performance improvements later as the fatigue induced may temporarily mask performance gains. In the context of a competitive season, this may be considered a desirable outcome as the benefits of preseason training will consequently last deeper into the season. 

The presence of high volumes of concurrent training and other constraints associated with high level sport limited the controls that could be placed on the study design. This, in turn, makes it challenging to precisely determine the isolated impact of the two training structures. It would be of scientific value to explore an analogous question in a more controlled setting. Nonetheless, the applied design of this methodology has increased ecological validity and is therefore of considerable value to practitioners in the field. It should also be noted that this investigation contained a small sample size, making interpretation of the observed effects less certain. Finally, future research should include more comprehensive testing that includes measures of maximal strength and mid-testing protocols to properly elucidate the time course of changes in both maximal strength and high velocity expressions of force throughout training. 

From a practical perspective, these findings demonstrate the challenges in developing explosive strength characteristics in the presence of high volumes of concurrent training. Sport scientists should note that even small changes in a sequenced strength-power training plan during this period will result in practically different changes in CMJ performance metrics. If coaching staff decide that maximizing high velocity capabilities earlier in the season (or for a given event) is the priority, then a focused sequenced strategy may be preferable. However, a more mixed methods sequenced approach might enable improvements in explosive capabilities to be realised later into the season. It is also important for practitioners to consider the training age and existing physical qualities when making such training decisions. 

## 5. Conclusions

Superior improvements in the timing of force production during the CMJ occur when a greater emphasis is placed on the targeted strength attribute in each block of a sequenced strength training plan during an elite junior Australian football preseason. The greater use of heavier loads in the first block likely enabled an enhanced adaptive response to the high velocity training in block 2 despite the high volume of concurrent training experienced by the players. However, it is possible that performance gains in the mixed group may not be realised until later in the season. Although these outcomes are highly applicable in the field, we encourage future research to build on these findings in a more controlled setting.

## Figures and Tables

**Table 1:** Resistance training plan for both the Focused and Mixed groups for the first mesocycle. BB: barbell; BM: body mass; DB: dumbbell; RIR: Estimated repetitions in reserve; SA: single arm. The manipulated lifts are indicated in bold. Where a range for sets or reps are presented, players progressed from the lower end to the higher end over the first mesocycle (weeks 1−4).

	Focused					Mixed				
**Week 1–4**	Day 1					Day 1				
		Lift	Sets	Reps	Loading		Lift	Sets	Reps	Loading
		**Back squat**	**3–4**	**6**	**75–80% 1 RM**		**Back squat**	**3–4**	**6**	**75–80% 1 RM**
		**Split squat**	**3–4**	**4**	**2 RIR**		**Jump squat**	**3–4**	**4**	**50% BM**
		Bench press	3–4	5	80–85% 1 RM		Bench press	3–4	5	80–85% 1 RM
		Chest pass	3–4	8	8kg MB		Chest pass	3–4	8	8kg MB
		Pull up	3	8–10	BM +		Pull up	3	8–10	BM+
	Day 2					Day 2				
		**Front squats**	**3–4**	**5**	**2 RIR**		**Front squats**	**3–4**	**5**	**2 RIR**
		**Countermovement shrug**	**3–4**	**5**	**75–80% BM**		**Jump shrug**	**3–4**	**5**	**30% BM**
		Inverted row	4	8–10	2 RIR		Inverted row	4	8–10	2 RIR
		DB shoulder press	4	8	2 RIR		DB shoulder press	4	8	2 RIR

**Table 2:** Resistance training plan for both the Focused and Mixed groups for the second mesocycle. BB: barbell; BM: body mass; DB: dumbbell; RIR: Estimated repetitions in reserve; SA: single arm. The manipulated lifts are indicated in bold. Training was reduced by one set across all exercises in week 8.

	Focused					Mixed				
**Weeks 5–8**	Day 1					Day 1				
		**Jump squat**	**4**	**6**	**50% BM**		**Back squat**	**4**	**3**	**2 RIR**
		**Plyo lunge**	**4**	**5**	**BM**		**Pylo lunge**	**4**	**5**	**BM**
		Chin ups	4	5	BM		Chin ups	4	6	BM+
		SA DB chest press	4	5	2 RIR		SA DB chest press	4	5	2 RIR
		BB bent over row	3	10	2 RIR		BB bent over row	3	10	2 RIR
	Day 2					Day 2				
		**Jump shrug**	**4**	**6**	**50% BM**		**Front squat**	**4**	**3–4**	**2 RIR**
		**Broad jump**	**4**	**5**	**BM**		**Drop jump**	**4**	**5**	**BM**
		Banded push up	4	8	Red Band		Banded push up	4	8	Red Band
		Inverted row	4	8	2 RIR		Inverted row	4	8	BW

**Table 3:** Comparison of the unloaded countermovement jump (CMJ) variables before and after both training interventions. Pre-post values are presented as mean ± SD.

CMJ Unloaded	Pre	Post	% ∆	*P*
Focused				
Flight Time:Contraction Time	0.65 ± 0.09	0.68 ± 0.06	10.0	0.18
Contraction Time (ms)	863 ± 132	808 ± 87	−9.8	0.15
Peak Velocity (m/s)	2.76 ± 0.11	2.74 ± 0.12	−0.2	0.61
Eccentric Mean Braking Force (N/kg)	11.50 ± 0.49	11.68 ± 0.46	6.5	0.20
Eccentric Duration (ms)	580 ± 95	536 ± 58	−15.3	0.13
Concentric Mean Force (N/kg)	19.29 ± 1.47	19.59 ± 1.19	−2.3	0.39
Concentric Duration (ms)	283 ± 41	271 ± 32	1.3	0.22
Mixed				
Flight Time:Contraction Time	0.61 ± 0.07	0.60 ± 0.08	−1.6	0.71
Contraction Time (ms)	896 ± 64	911 ± 68	1.7	0.63
Peak Velocity (m/s)	2.76 ± 0.15	2.76 ± 0.16	0.0	1.00
Eccentric Mean Braking Force (N/kg)	11.69 ± 0.69	11.52 ± 0.31	−1.4	0.51
Eccentric Duration (ms)	605 ± 48	606 ± 56	0.1	0.96
Concentric Mean Force (N/kg)	18.93 ± 1.44	18.54 ± 1.35	−2.1	0.38
Concentric Duration (ms)	291 ± 32	305 ± 29	4.8	0.30

**Table 4:** Comparison of the countermovement jump (CMJ) + 20 kg variables before and after both training interventions. Pre-post values are presented as mean ± SD.

CMJ + 20 kg	Pre	Post	% ∆	*P*
Focused				
Flight Time:Contraction Time	0.41 ± 0.06	0.43 ± 0.05	4.7	0.22
Contraction Time (ms)	1067 ± 172	1049 ± 113	−1.7	0.67
Peak Velocity (m/s)	2.30 ± 0.15	2.33 ± 0.12	1.5	0.25
Eccentric Mean Braking Force (N/kg)	13.76 ± 0.56	13.71 ± 0.42	−0.4	0.82
Eccentric Duration (ms)	708 ± 109	687 ± 69	−3.0	0.48
Concentric Mean Force (N/kg)	19.80 ± 1.37	19.80 ± 1.28	0.0	1.00
Concentric Duration (ms)	359 ± 65	362 ± 48	0.9	0.83
Mixed				
Flight Time:Contraction Time	0.40 ± 0.06	0.40 ± 0.04	−0.7	0.88
Contraction Time (ms)	1099 ± 89	1107 ± 64	0.7	0.86
Peak Velocity (m/s)	2.29 ± 0.17	2.34 ± 0.15	2.0	0.20
Eccentric Mean Braking Force (N/kg)	13.90 ± 0.49	13.61 ± 0.18	−2.0	0.25
Eccentric Duration (ms)	714 ± 65	717 ± 32	0.4	0.92
Concentric Mean Force (N/kg)	19.32 ± 1.15	19.35 ± 0.96	0.2	0.91
Concentric Duration (ms)	385 ± 37	390 ± 38	1.2	0.77
